# The Treatment of Incipient Insanity

**Published:** 1905-03-11

**Authors:** 


### THE TREATMENT OF INCIPIENT INSANITY.

Custom is a good servant but a bad master, and its power is nowhere more in evidence than in the hidebound attitude both of the public and the law towards the psychoses. To them the affections of the mind, mysterious as we must acknowledge them to be, are so mysterious as to be beyond the application of considerations adapted to more corporeal maladies. Otherwise, as was pointed out by Sir John Batty Tuke before the Neurological Society, there would not exist, as unfortunately there does, a disinclination to afford the insanities the early treatment which is recognised as the essential of success in all other forms of disease. At present no case of insanity, except among the moneyed classes, can be submitted to really suitable treatment until the condition is sufficiently far advanced to warrant certification; until, that is to say, the most invaluable epoch of the disease has been allowed to slip away. What is required, said the speaker, is, in the first place, an increase in the number of Lunacy Commissioners, and in the second place institutions for the reception of early cases of mental disease among the poor. He quoted the state of affairs that obtains in Germany. In that country there are attached to each of the 20 universities clinics of psychiatry under the superintendence of a professor. Special accommodation is provided, and through these clinics pass all persons reputed to be of unsound mind. Inveterate cases are handed on to the asylums, but all slighter and more hopeful cases are treated to a termination in these clinics—cases, for instance, such as neurasthenia, or insanity following fevers or operations. These are not reported to the State Office at all.

It appears that in this country Glasgow has the credit for the first departure in this direction, and the experiment has been thoroughly successful. It remains for the profession to educate public opinion in the knowledge that insanity, like other diseases, is capable of rational treatment; that the first essential of all successful treatment is its application before the condition to be dealt with has become inveterate; and that it is better economy to prevent an establishment of insanity, and to retain a good citizen, than to make adequate provision for a useless member of the community after sufficient pains have been taken to render him permanently useless.

